# Accuracy of Anatomical Depictions in Cone Beam Computed Tomography (CBCT)-Reconstructed Panoramic Projections Compared to Conventional Panoramic Radiographs: A Clinical Risk-Benefit Analysis

**DOI:** 10.7759/cureus.44723

**Published:** 2023-09-05

**Authors:** Aniket Jadhav, Neha G Desai, Aditya Tadinada

**Affiliations:** 1 Oral and Maxillofacial Radiology, Virginia Commonwealth University School of Dentistry, Richmond, USA; 2 Dentistry and Public Health, Jefferson Dental Care (JDC) Healthcare, Houston, USA; 3 Oral and Maxillofacial Radiology, University of Connecticut, Farmington, USA

**Keywords:** radiographic diagnosis, dental anatomy, panoramic radiograph, dental radiology, cone-beam computed tomography (cbct)

## Abstract

Introduction: Two-dimensional (2D) radiographs are the standard of care for diagnosis and treatment planning in the day-to-day practice of dentistry. With the growing popularity of cone beam computed tomography (CBCT), it is now becoming the standard of care in many areas of general dentistry due to its ability to create non-linear projections from volumetric data. The CBCT-generated non-orthogonal radiographs can serve as easy-to-use 2D and three-dimensional (3D) diagnostic tools and offer a similar experience for diagnosis as conventional 2D images. The aim of this study is to compare the accuracy of conventional radiographs and CBCT-generated projections to identify relevant anatomic landmarks and their associated variants.

Methods: Thirty-two patients referred to the University of Connecticut School of Dental Medicine’s Advanced Imaging Center were selected for this retrospective analysis. Nineteen anatomical landmarks were retrospectively assessed on conventional panoramic and CBCT scans generated panoramic radiographs using two different digital imaging and communications in medicine viewers. A total of 1,216 anatomical landmarks were evaluated by two oral and maxillofacial radiologists to assess the accuracy and consistency of the depiction of radiographic anatomy.

Results: There was a very good agreement between the two evaluators with a Cohen's kappa value of 0.934. McNemar change test concluded that the anatomical assessment values compared between conventional panoramic and CBCT-generated panoramic radiographs are similar.

Conclusion: This study showed that CBCT-generated panoramic images are comparable to conventional panoramic radiographs in identifying anatomical landmarks typically evaluated using a conventional panoramic projection. In addition, they have the added advantage of having 3D information in the acquired volume to better evaluate the area of interest. In clinical situations where a mid- to large-volume CBCT scan is available, a simulated panoramic image can be generated using the CBCT volume, leaving exposure of the patient to the additional radiation of a panoramic image unnecessary.

## Introduction

Cone beam computed tomography (CBCT) is a valuable diagnostic tool for many oral and maxillofacial procedures and is increasingly being used by dental professionals for three-dimensional (3D) evaluations. Because this modality scans the maxillofacial skeleton in three dimensions, the derived reconstructed image can be used to visualize the area of interest in multiple projections [1]. Most, if not all, two-dimensional (2D) images traditionally used in dentistry can be simulated and reconstructed using post-processing algorithms [2]. Unlike the traditional images, the data acquired using CBCT scans can be refined to reduce or eliminate the error often encountered by conventional 2D images such as superimposition of anatomical structures, magnification, distortion, etc. [3]. Not exempt from these errors is the panoramic radiograph, one of the most widely used views in the maxillofacial region. It is the hypothesis of this cohort that CBCT-reconstructed panoramic projections will not present the distortions common to conventional panoramic imaging and will more accurately display the area of interest. The main objective of this study is to compare the accuracy of conventional panoramic radiographs and CBCT-generated panoramic image projection to identify relevant anatomic landmarks and their associated variants. To evaluate this, oral and maxillofacial radiologists were asked to identify anatomical landmarks in conventional panoramic images and CBCT reconstructed panoramic images using two CBCT programs that reconstruct the panoramic image using different reconstruction kernels.

Several important variables must be considered when the intent is to compare CBCT-reconstructed panoramic projection to a conventional panoramic radiograph. One of the key features would be to visualize and identify maxillofacial anatomy. It has been previously shown that CBCT scans are comparable to panoramic X-rays in this aspect [4]. In addition, the quality of CBCT radiographs being generated as slices will be compared to the 2D panoramic radiographs. Another key variable that should be addressed is radiation dosage. The radiation emitted by the two modalities will be compared and factored in when drawing conclusions.

## Materials and methods

Data collection

Thirty-two patients referred to the University of Connecticut School of Dental Medicine’s Advanced Imaging Center were selected for this retrospective analysis. All the CBCT scans and radiographic images selected for this study between 2010 to 2012 were de-identified, and all Health Insurance Portability and Accountability Act markers were removed from the image data sets. University of Connecticut Health Center Institutional Review Board issued approval 13-174-1 under exempt status.

The inclusion criteria for this study were patients with conventional panoramic images and CBCT scans with a field of view of nine inches or more and conventional panoramic images and CBCT scans with both the temporomandibular joints (TMJs) and inferior border of the mandible well visualized.

The conventional panoramic images were acquired using Sirona Orthophos XG digital panoramic machine (Dentsply Sirona, Charlotte, North Carolina, USA) with exposure parameters of 60 kvp and 7 mA.

**Figure 1:**
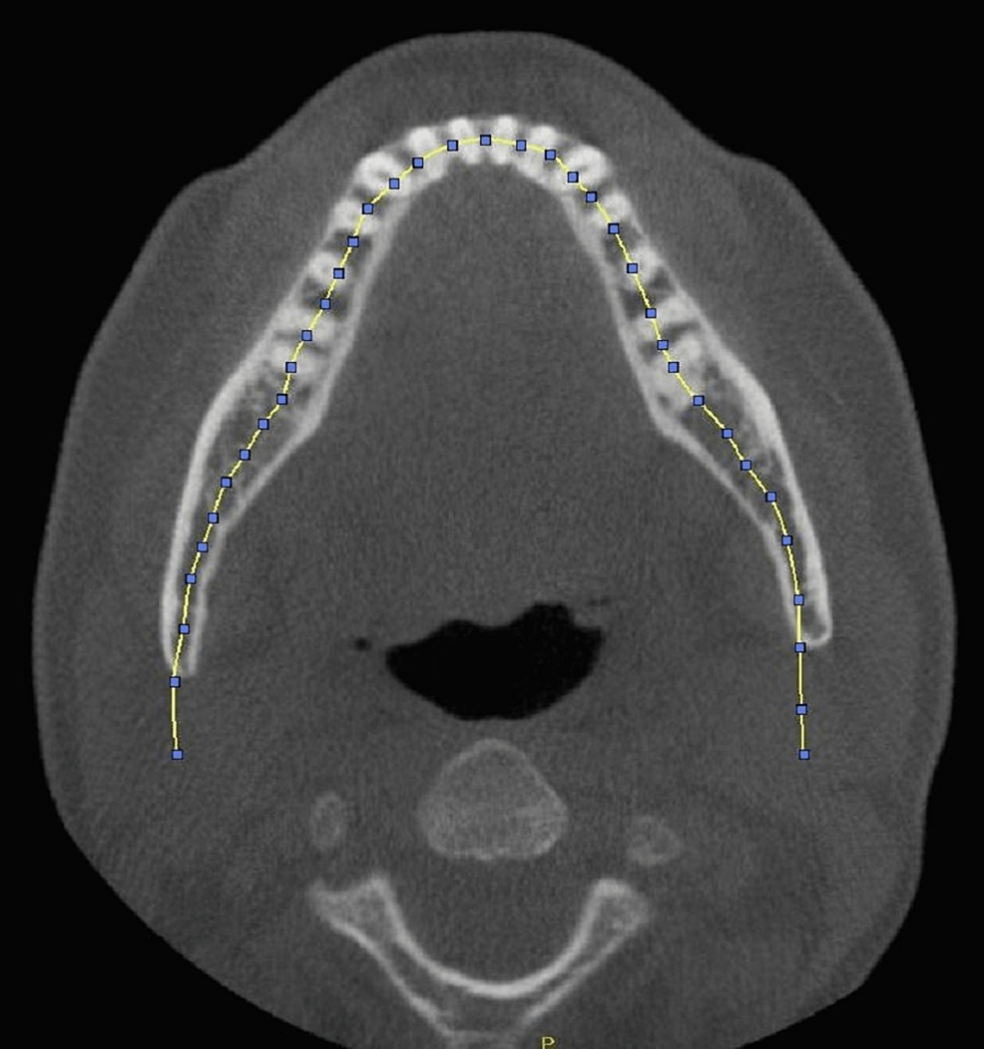
Focal trough generated by Software A reconstruction

**Figure 2:**
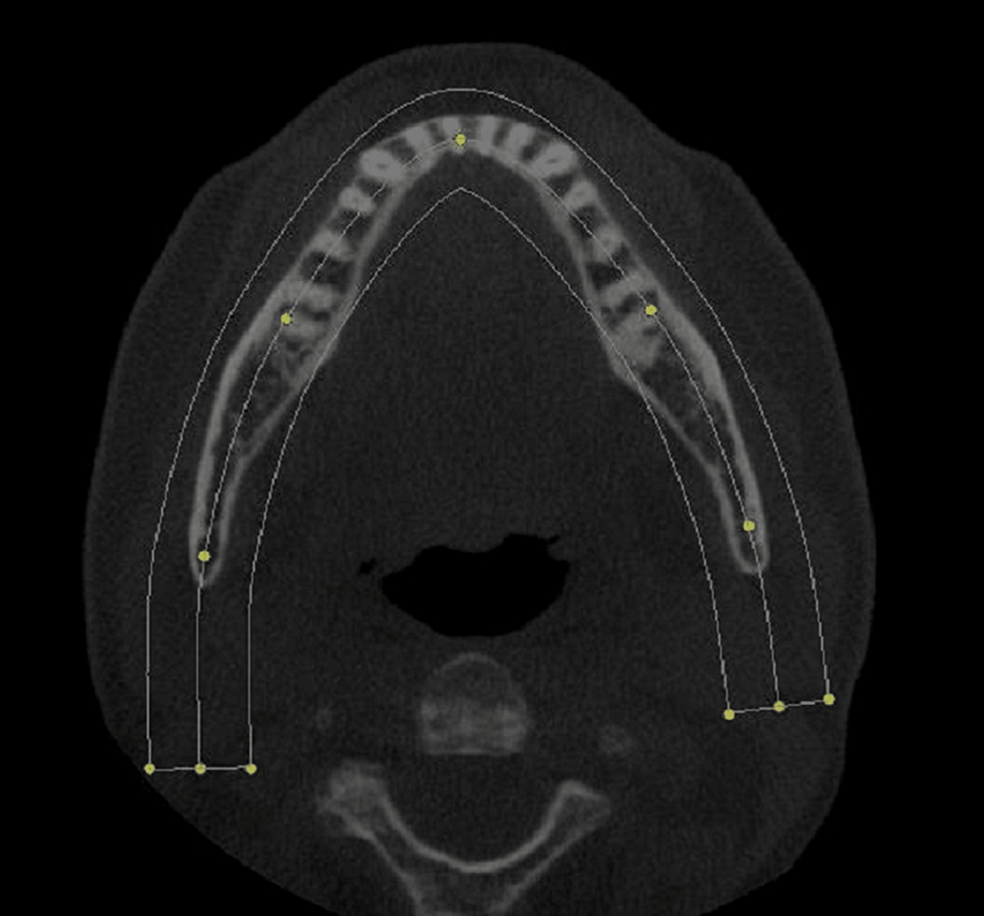
Focal trough generated by Software B reconstruction

Panoramic images from the CBCT data set acquired on Hitachi CB Mercury (Hitachi High-Tech Corporation, Tokyo, Japan) were generated using a CBCT reconstruction software program Invivo (Anatomage Inc, Sanjose, California, USA) which allows custom generation of the focal trough. Software programs CB Works 3.0 (Software A, Figure 1) and Invivo version 4 (Software B, Figure 2) were selected due to some of their important features. The software reconstructed program Invivo allows more flexibility for altering the thickness of the focal trough by accommodating the trough lines on the axial section, while the reconstructed CB Works 3.0 allows users to choose a preselected thickness size in mm.

It should be noted that CB Works 3.0 permits custom generation of the simulated panoramic, while the Invivo version 4 program only allows for thickness alteration of the focal trough, much like conventional panoramic imaging [5].

**Figure 3:**
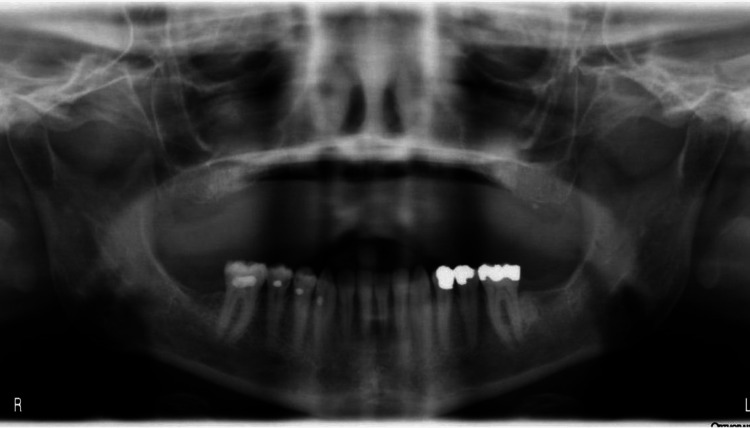
Conventional panoramic radiograph

**Figure 4:**
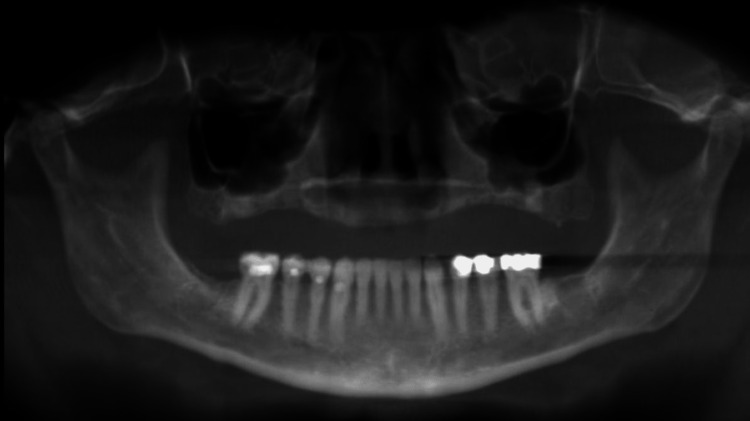
CBCT-reconstructed panoramic by Software A

**Figure 5:**
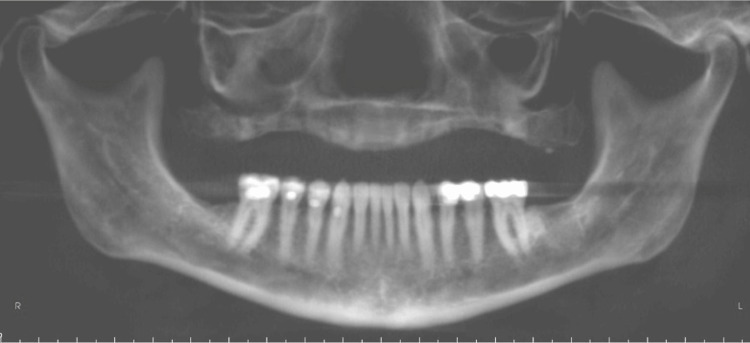
CBCT reconstructed panoramic by Software B

Simulated panoramic image projections were generated using both software programs. Examples of the three modalities can be seen below. Figure 3 shows an example of a conventional panoramic X-ray. Figures 4-5 show CBCT-reconstructed panoramic images using CB Works 3.0 and Invivo version 4, respectively.

Analysis of the images

A total of 1,216 anatomical landmarks were evaluated from 32 patients using conventional and CBCT-generated panoramic images. A total of 96 panoramic images were analyzed from 32 patients. All these images were randomized and presented to the examiners (AT and AJ) for the identification of typical 19 anatomical landmarks (Table 1). Two oral and maxillofacial radiologists (AT and AJ) evaluated the anatomical landmarks using a two-point scale of agreement, which measured the consistency of identification of these anatomical landmarks. The data was statistically analyzed to evaluate consistency between the raters, the imaging modalities, and the reconstruction software programs in their ability to identify pertinent anatomical landmarks.

**Table 1:** Anatomical landmarks evaluated on three panoramic projections

Anatomical landmarks
Mandibular condyle
Glenoid fossa
Articular eminence
Zygomatic arch
Zygomatic process of maxilla
Zygoma
Sigmoid notch
Coronoid process
Mandibular ramus
Angle of mandible
Inferior border of the mandible
Mandibular canal
Mental foramina
Lateral orbital rim
Medial wall of the maxillary sinus
Postero-lateral wall of the maxillary sinus
Floor of the maxillary sinus
Cervical spine
Dentoalveolar structures

Statistical analysis

Cohen’s kappa is a well-established measurement for determining inter-operator reliability when dichotomous outcomes are considered [6]. In our study too, all the images were evaluated by two oral and maxillofacial radiologists, and intraoperator reliability was measured using Cohen’s kappa analysis. The McNemar test has been successfully used in the literature to compare the differences in two sets of observations [7,8]. We also used the McNemar Change test to assess differences in the diagnostic ability of conventional and CBCT-reconstructed panoramic images.

## Results

Table 2 shows the Cohen’s kappa results. As you can see from the results, the kappa value is 0.934. It signifies that the strength of agreement is “very good.” This further underlines the strong inter-operator reliability in this study.

**Table 2:** Cohen’s kappa for intra-operator reliability

Symmetric measures					
		Value	Asymp. Std. Error^a^	Approx. T^b^	Approx. Sig.
Measure of agreement	Kappa	0.934	0.025	32.568	0.000
N of valid cases		1216			

All landmarks were easily identified by examiners in the conventional panoramic X-ray images. Table 3 shows the preference of the examiners for the two software programs. A value of 1 signifies a positive choice. As seen in Table 3, it is clear that both the observers, AT and AT, preferred reconstruction Software B over reconstruction Software A.

**Table 3:** Subjective preference of CBCT-reconstruction software program

Examiner	Reconstruction Software A	Reconstruction Software B
Examiner 1	-	1
Examiner 2	-	1

**Figure 6:**
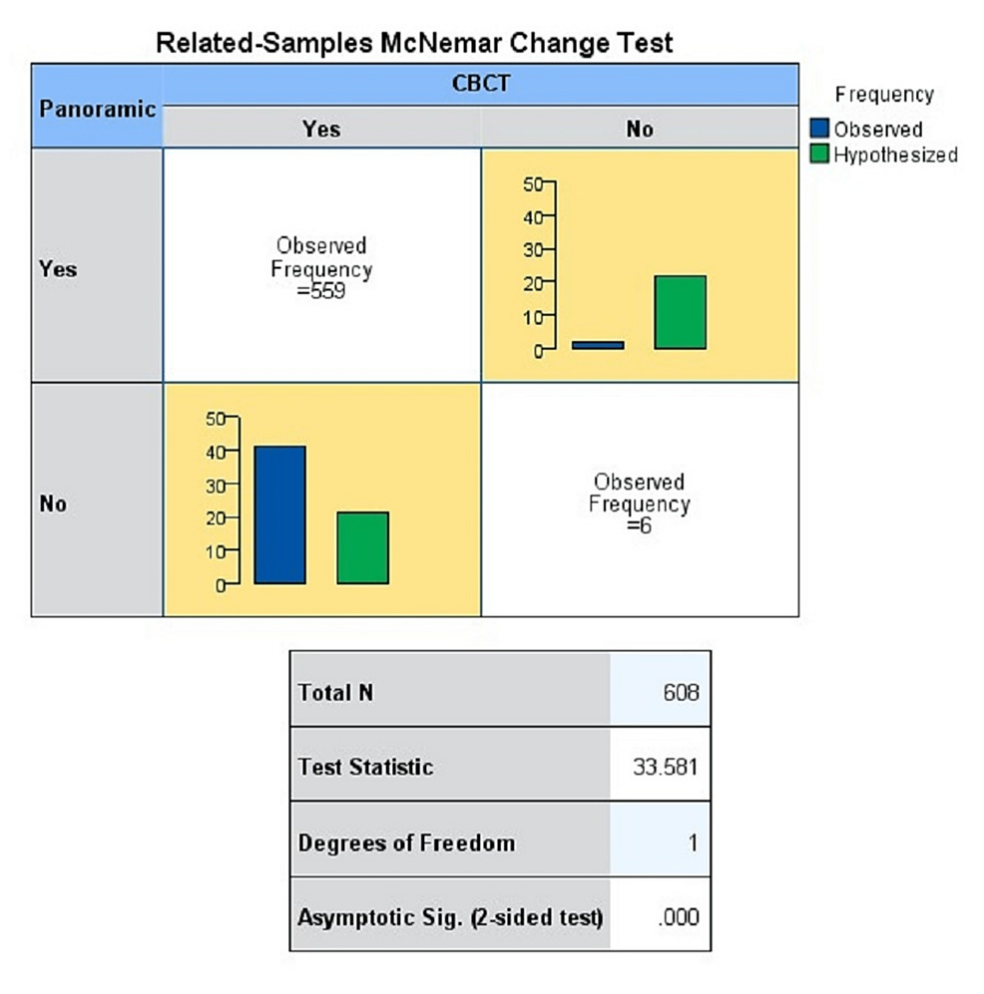
McNemar change test

For the McNemar change test, the null hypothesis was that there are different values across conventional and CBCT-generated panoramic images. However, as observed from the test, the obtained chi-square value is greater than the critical chi-square value. Thus, the null hypothesis is rejected and it can be concluded that the values obtained from conventional and CBCT-generated panoramic images are similar (Table 4, Figure 6).

**Table 4:** McNemar change test

Hypothesis test summary				
	Null hypothesis	Test	Sig.	Decision
1	The distributions of different values across panoramic and CBCT are equally likely	Related-sample McNemar test	0.000	Reject the null hypothesis
Asymptotic significances are displayed. The significance level is 0.05.				

## Discussion

Panoramic imaging has been the modality of choice for the assessment of the maxillofacial skeleton in the field of dentistry for a long time [9]. Conventional panoramic images are easy to acquire, affordable, and provide a concise overview of maxillofacial anatomy for diagnostic evaluation and treatment planning. CBCT technology has gained popularity among dental professionals and becoming the preferred modality of choice for evaluating the maxillofacial anatomy in three dimensions [10]. CBCT data allows users to reconstruct panoramic images by drawing a customized focal trough using proprietary software programs [11].

In this retrospective analysis, we observed a high level of inter- and intra-operator reliability, measured using Cohen's kappa statistic. Cohen’s kappa statistic is commonly used to measure inter- and intra-observer agreement in other dental radiological studies [12,13]. High internal consistency (0.98) was achieved for all three types of panoramic images. All 19 anatomical landmarks were easy to identify on the conventional panoramic images, while 18 anatomical landmarks were easy to identify on the simulated panoramic images. One explanation for this difficulty in identification is the use of a thick focal trough to compensate for skeletal malocclusion and derive images that closely simulate a conventional panoramic radiograph. It is observed that the identification of different anatomical structures depends on their position with respect to the focal trough and, thus, can lead to magnification or distortion of anatomical landmarks rendering them difficult to determine on panoramic radiographs [14,15]. However, in such cases, the focal trough can be drawn on the mandible so that the simulated panoramic image will project the inferior alveolar canal more precisely. When asked to provide a subjective evaluation for each of the reconstruction software programs used in the study, both examiners preferred Software B over Software A.

When examiners were asked to identify the anatomical landmarks, they were examining only a static simulated image projection. A major advantage of CBCT is the use of cross-sectional image “slices,” allowing the examiner to vary the depth at which they are visualizing the structures. This advantage makes tasks such as the detection of pathology or measurement of root depth much more accurate [16]. It is the opinion of this cohort that if the examiner were allowed to manipulate the images as the software programs are allowed, then the ability to visualize the anatomy would significantly increase the ability for accurate diagnosis and treatment planning.

An important consideration in the comparison of these two imaging modalities is the concept of radiation dose. It is a well-established fact that 3D imaging yields a greater amount of diagnostic information than 2D imaging. However, the risk to the patient cannot be increased unnecessarily without a higher diagnostic yield. The increase in radiation dose may lead to chronic side effects. It is important for dentists to adopt methods leading to the prevention and control of chronic health conditions in patients [17]. In a conventional panoramic X-ray, the dose of radiation is typically about 30 microsieverts depending on the panoramic machine [18]. In CBCT imaging, the dose greatly varies based on the operator’s choice of variable such as field of view and voltage, and ranges from 68 microsieverts to many hundreds more [19]. The choice as to whether this additional dose is necessary would need to be made by the clinician, although if the patient already has a CBCT scan, then additional conventional panoramic image may not yield any more diagnostic information than already available which can be further utilized by specific image reconstructions like the panoramic.

As a final point of discussion, this cohort would like to address the subjective choice of software programs by the examiners. Both examiners chose the Invivo software reconstruction program. As was previously stated, this program uses a focal trough much like a conventional panoramic radiograph. It is the opinion of this study group that the examiners preferred this view because it more closely resembled the conventional panoramic radiographs that have been a staple in their practice for many years. This concept of familiarity could contribute to the results that have been seen in this study.

Lastly, we acknowledge the limitations of the study, especially the absence of a comparison between images showing patient motion during conventional panoramic and CBCT scan acquisitions. Patient motion during CBCT scan can lead to blurriness of the anatomy that can reflected in reconstructed panoramic radiographs. While motion in conventional panoramic projections can result in more distorted images compared to CBCT-generated panoramic images, this comparison is crucial as both imaging techniques are susceptible to motion artifacts due to exposure time and patient positioning. Another limitation of the study pertains to image distortion observed in CBCT-reconstructed panoramic projections due to the extension of the focal trough toward the oropharynx. Consequently, we did not compare airspaces between the two imaging modalities.

Another limitation of this study design is not having a general dentist as an evaluator. The CBCT-generated panoramic projection may appear familiar to oral and maxillofacial radiologists but may appear different in perception as well as for assessment to the general dentist. Unlike panoramic X-rays, which are a 2D imaging modality, a practitioner can delve deeper into the anatomy of a patient using CBCT. If the dental professional needed additional information about a patient’s mouth, patients would not need to be exposed to additional radiation if a CBCT scan had been done. The 3D aspect allows for the manipulation of the image to get a more accurate diagnostic picture, but the radiation exposure to the patient in CBCT is significantly higher for the field of view that captures maxillofacial anatomy including TMJs when compared to conventional panoramic X-rays. If the clinical situation justifies the use of CBCT in the first place that covers the maxillofacial region, then the additional conventional panoramic exposure is not warranted.

## Conclusions

This study has shown that CBCT-generated panoramic images are comparable to conventional panoramic radiographs in identifying anatomical landmarks typically evaluated using a conventional panoramic projection. In addition, they have the added advantage of having 3D information in the acquired volume to better evaluate the area of interest. The study is certainly limited by the number of patients and technical factors of CBCT that may affect the diagnostic ability of CBCT-reconstructed panoramic images. Visibility of the anatomy is limited by the field of view of the CBCT scan, and, hence, not all CBCT with limited view may offer a comparable view of maxillofacial anatomy. In clinical situations where a mid- to large-volume CBCT scan is available, a simulated panoramic image can be generated using the CBCT volume, leaving exposure of the patient to the additional radiation of a panoramic image unnecessary.

The main objective of this study was to compare the accuracy of conventional panoramic radiographs and CBCT-generated panoramic image projection to identify relevant anatomic landmarks and their variants. This objective was achieved within the limitations of the study design as discussed. The results of this study pointed out that the two modalities are comparable in their diagnostic abilities, although future investigation is needed to assess the effects of CBCT artifacts such as beam hardening, noise, and motion on the reconstructed panoramic radiographs and their diagnostics ability.
